# Surface roughness, optical properties, and microhardness of additively and subtractively manufactured CAD‐CAM materials after brushing and coffee thermal cycling

**DOI:** 10.1111/jopr.13796

**Published:** 2023-11-27

**Authors:** Gülce Çakmak, Mustafa Borga Donmez, Marcella Silva de Paula, Canan Akay, Manrique Fonseca, Çiğdem Kahveci, Samir Abou‐Ayash, Burak Yilmaz

**Affiliations:** ^1^ Department of Reconstructive Dentistry and Gerodontology School of Dental Medicine University of Bern Bern Switzerland; ^2^ Department of Prosthodontics Faculty of Dentistry Istinye Univeristy İstanbul Turkey; ^3^ Department of Prevention and Oral Rehabilitation Universidade Federal de Goiás Goiánia GO Brazil; ^4^ Department of Prosthodontics Faculty of Dentistry Eskisehir Osmangazi Univeristy Eskisehir Turkey; ^5^ Translational Medicine Research and Clinical Center Osmangazi University Eskisehir Turkey; ^6^ Ordu Oral and Dental Health Center Ordu Turkey; ^7^ Department of Restorative Preventive and Pediatric Dentistry School of Dental Medicine University of Bern Bern Switzerland; ^8^ Division of Restorative and Prosthetic Dentistry The Ohio State University Columbus Ohio USA

**Keywords:** additive manufacturing, brushing, coffee thermal cycling, roughness, stainability, translucency

## Abstract

**Purpose:**

To evaluate the surface roughness, optical properties, and microhardness of additively or subtractively manufactured CAD‐CAM materials after simulated brushing and coffee thermal cycling.

**Material and methods:**

Two additively manufactured resins (Crowntec, CT and VarseoSmile Crown Plus, VS) and 3 subtractively manufactured materials (a reinforced composite (Brilliant Crios, BC), a polymer‐infiltrated ceramic network (Enamic, VE), and a feldspathic ceramic (Mark II, VM)) were used to fabricate disk‐shaped specimens (Ø10×1‐mm) (*n* = 10). Surface roughness, Vickers microhardness, and color coordinates were measured after polishing, while surface roughness was also measured before polishing. Specimens were then subjected to 25000 cycles of brushing and 10000 cycles of coffee thermal cycling, and measurements were repeated after each time interval. Color difference (ΔE_00_) and relative translucency parameter (RTP) were calculated. Robust analysis of variance test was used to evaluate surface roughness, ΔE_00_, and RTP data, while generalized linear model analysis was used for microhardness data (α = 0.05).

**Results:**

Material type and time interval interaction affected tested parameters (*p* ≤ 0.002). In addition, material type affected all parameters (*p* < 0.001) other than surface roughness (*p* = 0.051), and time interval affected surface roughness and microhardness values (*p* < 0.001). Tested materials mostly had their highest surface roughness before polishing (*p* ≤ 0.026); however, there was no clear trend regarding the roughness of materials within different time intervals along with ΔE00 and RTP values within materials or time intervals. VS and CT had the lowest microhardness regardless of the time interval, while the remaining materials were listed as VM, VE, and BC in decreasing order (*p* < 0.001). Coffee thermal cycling only reduced the microhardness of VM (*p* < 0.001).

**Conclusions:**

Tested additively manufactured resins can be considered more susceptible to simulated brushing and coffee thermal cycling than the other materials, given the fact that their surface roughness and ΔE00 values were higher than previously reported acceptability thresholds and because they had the lowest microhardness after all procedures were complete.

Advancements in computer‐aided design and computer‐aided manufacturing (CAD‐CAM) technologies have enabled the use of restorative materials in different chemical compositions and can be used monolithically.^1^, ^2^ Glass ceramics and composite resins are among those materials^3^, ^4^ and present with advantages and disadvantages.^5^ An alternative to those materials has been a polymer‐infiltrated ceramic network, which combines the advantages of both ceramics and composite resins^6^, ^7^, ^8^ due to its unique chemical composition of urethane dimethacrylate and triethylene glycol dimethacrylate infiltrated into feldspathic ceramic.^9^, ^10^, ^11^, ^12^, ^13^

Subtractive manufacturing has been the dominant fabrication method for CAD‐CAM restorations in the last decade.^14^ However, with its increasing popularity, additive manufacturing is now an alternative.^15^, ^16^ This technology is based on layer‐by‐layer construction, which allows the fabrication of products with complex geometries.^17^ Different materials can be processed with additive manufacturing,^18^, ^19^, ^20^, ^21^, ^22^ and in recent years, additively manufactured resins indicated for definitive restorations have also been marketed.^14^, ^15^, ^23^ Some of these resins are referred to as composites (Crowntec; Saremco Dental AG)^24^ and some are referred to as hybrid composites (VarseoSmile Crown Plus; Bego).^23^, ^25^

Regardless of the manufacturing method used, a restorative material must be able to maintain its optical and mechanical properties throughout its clinical service. However, a restoration's surface may deteriorate intraorally, due to the temperature changes, staining solutions, and brushing.^5^, ^26^ Even though previous studies investigated additively manufactured composite resins,^14^, ^15^, ^16^, ^18^, ^19^, ^20^, ^21^, ^27^, ^28^, ^29^ the knowledge on the effect of simulated brushing and coffee thermal cycling on these materials, particularly for those indicated for definitive restorations, is lacking. Clinicians could comprehend the limitations of these materials by using the findings of studies that investigate their properties against materials of similar composition in different situations. In addition, making comparisons with commonly used feldspathic ceramic, which is also indicated for the same treatment options, under standardized conditions would elaborate the knowledge of their applicability. Therefore, the present study aimed to compare the surface roughness, stainability, translucency, and microhardness of different CAD‐CAM materials fabricated either by using additive (a composite resin and a hybrid composite resin) or subtractive (a reinforced composite, a polymer‐infiltrated ceramic network, and a feldspathic ceramic) manufacturing after simulated brushing and coffee thermal cycling. The null hypotheses were that (i) the surface roughness of tested CAD‐CAM materials would not be affected by material type and time interval, (ii) the stainability of tested CAD‐CAM materials would not be affected by material type and time interval, (iii) the translucency of tested CAD‐CAM materials would not be affected by material type and time interval, and (iv) microhardness of tested CAD‐CAM materials would not be affected by material type and time interval.

## MATERIAL AND METHODS

**FIGURE 1:**
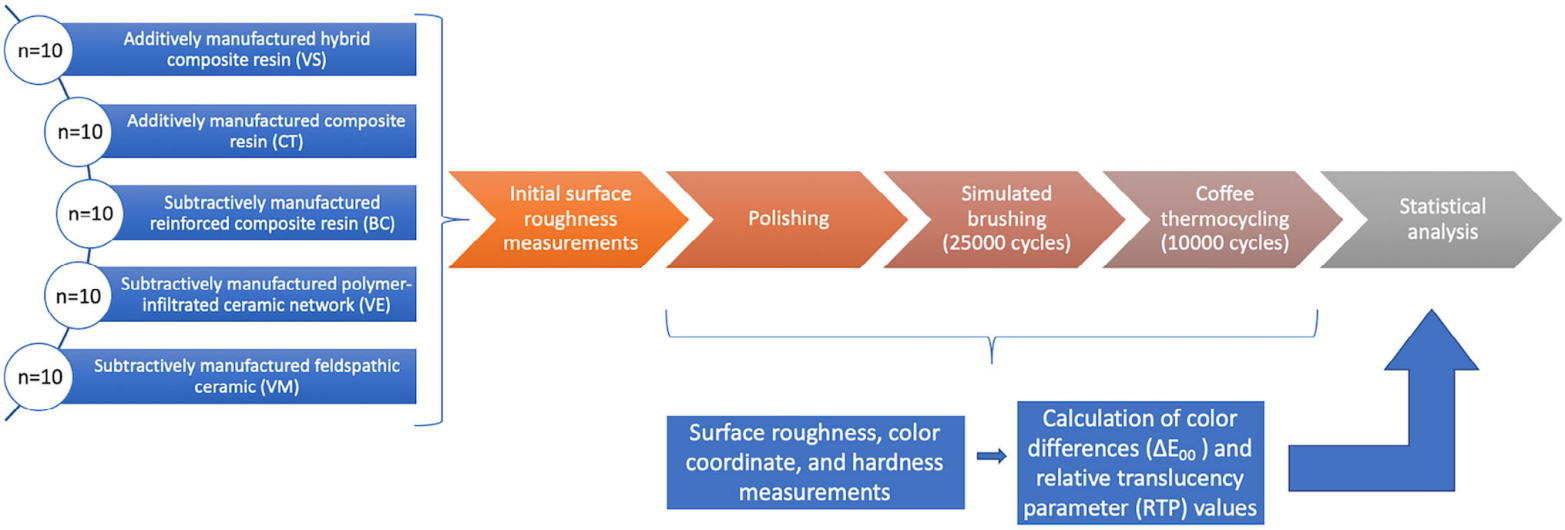
Overview of this study. BC: BRILLIANT Crios; CT: CROWNTEC; VE: enamic; VM: Mark II, VS: VarseoSmile Crown Plus.

In line with previous studies on the roughness, optical properties, and microhardness of CAD‐CAM materials after brushing or coffee thermal cycling,^6^, ^9^, ^30^, ^31^, ^32^ 10 disk‐shaped specimens were fabricated from each of the tested additively manufactured resins (Crowntec; Saremco Dental AG [CT] and VarseoSmile Crown Plus; Bego [VS]) and subtractively manufactured CAD‐CAM materials (a reinforced composite (Brilliant Crios; Coltène AG [BC]), a polymer‐infiltrated ceramic network (Enamic; Vita Zahnfabrik [VE]), and a feldspathic ceramic (Mark II; Vita Zahnfabrik [VM]) in A1 shade. The chemical compositions of the materials are given in Table 1. Figure 1 shows the overview of the present study.

**TABLE 1:** Materials used in this study.

Material	Type	Composition
VarseoSmile Crown Plus (VS)	Additively manufactured hybrid composite resin	Esterification products of 4,4’‐isopropylidiphenol, ethoxylated and 2‐methylprop‐2enoic acid, silanized dental glass, methyl benzoylformate, diphenyl (2,4,6‐trimethylbenzoyl) phosphine oxide, 30−50 wt%—inorganic fillers (particle size 0.7 μm)
CROWNTEC (CT)	Additively manufactured composite resin	Esterification products of 4,4’‐isopropylidiphenol, ethoxylated and 2‐methylprop‐2enoic acid, silanized dental glass, pyrogenic silica, initiators. Total content of inorganic fillers (particle size 0.7 μm) is 30 ‐ 50 wt%.
BRILLIANT Crios (BC)	Subtractively manufactured reinforced composite resin	70.7 wt% barium glass (<1 μm) and amorphous silica (SiO_2_; <20 nm), Cross‐linked methacrylates (Bis‐GMA, Bis‐EMA, TEGDMA)
Enamic (VE)	Subtractively manufactured polymer‐infiltrated ceramic network	14 wt% methacrylate polymer (UDMA, TEGDMA) and 86 wt% fine‐structure feldspathic ceramic network
Mark II (VM)	Subtractively manufactured feldspathic ceramic	>20 wt% feldspathic particles (average size of the particle 4 μm) 80 wt% of the glass‐matrix

For the fabrication of additively manufactured specimens (CT and VS), a disk‐shaped standard tessellation language (STL) file (Ø10×1‐mm) was designed with design software (Meshmixer v3.5.474; Autodesk Inc.). This STL file was transported into a nesting software (Composer v1.3.3; Asiga) and positioned on its flat surface. Supports were automatically generated and this configuration was duplicated 10 times. Specimens were printed with 50 μm layer thickness with a digital light processing (DLP) printer (MAX UV; Asiga). After fabrication, CT specimens were cleaned with an alcohol‐soaked (96%) cloth until all resin residues were completely removed, while VS specimens were ultrasonically cleaned in ethanol for 5 min (3 min of precleaning in reusable ethanol and an additional 2 min in fresh ethanol). Specimens were then air‐dried and light‐polymerized either with 4000 (CT, 2×2000) or 3000 (VS, 2×1500) light exposures (Otoflash G171; NK Optik) under a nitrogen oxide gas atmosphere.^24^, ^25^ For the fabrication of subtractively manufactured specimens (BC, VE, and VM), a 10 mm‐wide cylinder was designed in STL format by using the same software. This STL file was used to mill cylinders from CAD‐CAM blocks (PrograMill PM7; Ivoclar AG), which were then wet‐sliced into 1 mm‐thick specimens with a precision cutter (Vari/cut VC‐50; Leco Corp). All specimens were ultrasonically cleaned in distilled water for 10 min (Eltrosonic Ultracleaner 07–08; Eltrosonic GmbH) and dried with a paper towel before the measurements.

A non‐contact optical profilometer (FRT MicroProf 100, equipped with a CWL 300 μm sensor, resolution of 3 nm in z‐dimension; Fries Research & Technology GmbH)^30^ was used to record 6 linear traces (3 horizontal and 3 vertical) that had a length of 5.5 mm, a pixel density of 5501 point/line, and were 1 mm apart. Baseline surface roughness, in R_a_, of each trace was determined with the integrated software (Mark III, Fries Research & Technology GmbH, Gladbach, Germany) according to the International Organization for Standardization 4287 standard^33^ with a cutoff value (Lc) of 0.8 mm, and the average of these traces were calculated. After baseline surface roughness measurements, each set of specimens was polished by using the respective manufacturer's recommendations (Table 2), and surface roughness values were remeasured.

**TABLE 2:** Polishing methods used in this study.

Material	Polishing method
VarseoSmile Crown Plus (VS)	A slurry of coarse pumice in water (Pumice fine; Benco Dental) was used to conventionally polish one surface of all specimens for 90 seconds at 1500 rpm. Fine polishing was performed by using a polishing paste (Fabulustre; Grobet USA) for an additional 90 seconds
CROWNTEC (CT)	A slurry of coarse pumice in water (Pumice fine; Benco Dental) was used to conventionally polish one surface of all specimens for 90 seconds at 1500 rpm. Fine polishing was performed by using a polishing paste (Fabulustre; Grobet USA) for an additional 90 seconds
BRILLIANT Crios (BC)	Two‐step polishing kit (DIATECH Lab Finishing&Polishing Kit for BRILLIANT Crios; Coltène AG)
Enamic (VE)	Two‐step polishing kit (Vita Enamic Polishing Set; Vita Zahnfabrik)
VarseoSmile Crown Plus (VM)	Finishing with flexible discs (Sof‐Lex discs; 3 M ESPE) and high‐gloss polishing with a diamond polishing paste (VITA Polish Cera; Vita Zahnfabrik).

A digital spectrophotometer (CM‐26d; Konica Minolta),^30^, ^34^ which had a medium area view, 2‐degree human observer characteristics, and Commission International de I'Eclairage (CIE) D65 illumination was used to measure the color coordinates (L*, a*, and b*) defined by CIE on white, black, and gray backgrounds. A saturated sucrose solution with a refractive index of approximately 1.5 was used for the optical contact between specimens and background,^35^ and the spectrophotometer was calibrated before the measurement of each group. The sucrose solution was freshly prepared before measurements with a homogenizer (T25 digital Ultra Turrax; IKA). Three measurements were recorded on each background for each specimen and these values were averaged. All color measurements were performed by the same clinician (M.S.P.) in a temperature and humidity‐controlled room with daylight. In addition, the color coordinates of 4 unpolished specimens from each group were measured twice within 24 h to calculate the intrinsic error of the spectrophotometer.

A Vickers microhardness tester (M‐400 Hardness Tester; Leco Corp) was used to measure the initial microhardness values. Each specimen was subjected to a load of 980.7 mN for 10 s^36^ at 5 different sites that were at least 0.5 mm apart from each other. These values were then averaged to calculate the definitive microhardness value of each specimen.

After these measurements, specimens were subjected to 25000 cycles of artificial brushing (50000 strokes, each cycle considered as a linear back‐and‐forth brushing action at a frequency of 1.5 Hz) by using an automatic brushing machine (Bürstmaschine linear LR1; Syndicad Engineering) and FDA‐certified toothbrushes.^3^ Total brushing time of 25000 cycles (50000 strokes) was considered to replicate nearly 7 years as a period of 3650 cycles (7300 strokes) was assumed to simulate 1 year intraorally^31^, ^32^, ^37^ considering that a tooth surface is brushed 20 times a day. Six toothbrushes were mounted to the brushing machine with their bristles facing directly at the specimen surface. Deionized water and regular toothpaste (Nevadent Complex 3; DENTAL‐Kosmetik GmbH) were mixed to form a slurry in a 2:1 ratio by weight with a homogenizer (T25 digital Ultra Turrax; IKA) before testing.^38^, ^39^ Slurries were poured into each chamber of the brushing machine until the surface of the specimens was covered. The toothbrushes and slurry were changed with the new ones every 10000 cycles. A vertical load of 200 g was applied to each specimen during the horizontal movement of the brushes at room temperature (23°C). After brushing, the specimens were rinsed with distilled water and gently air‐dried.

**FIGURE 2:**
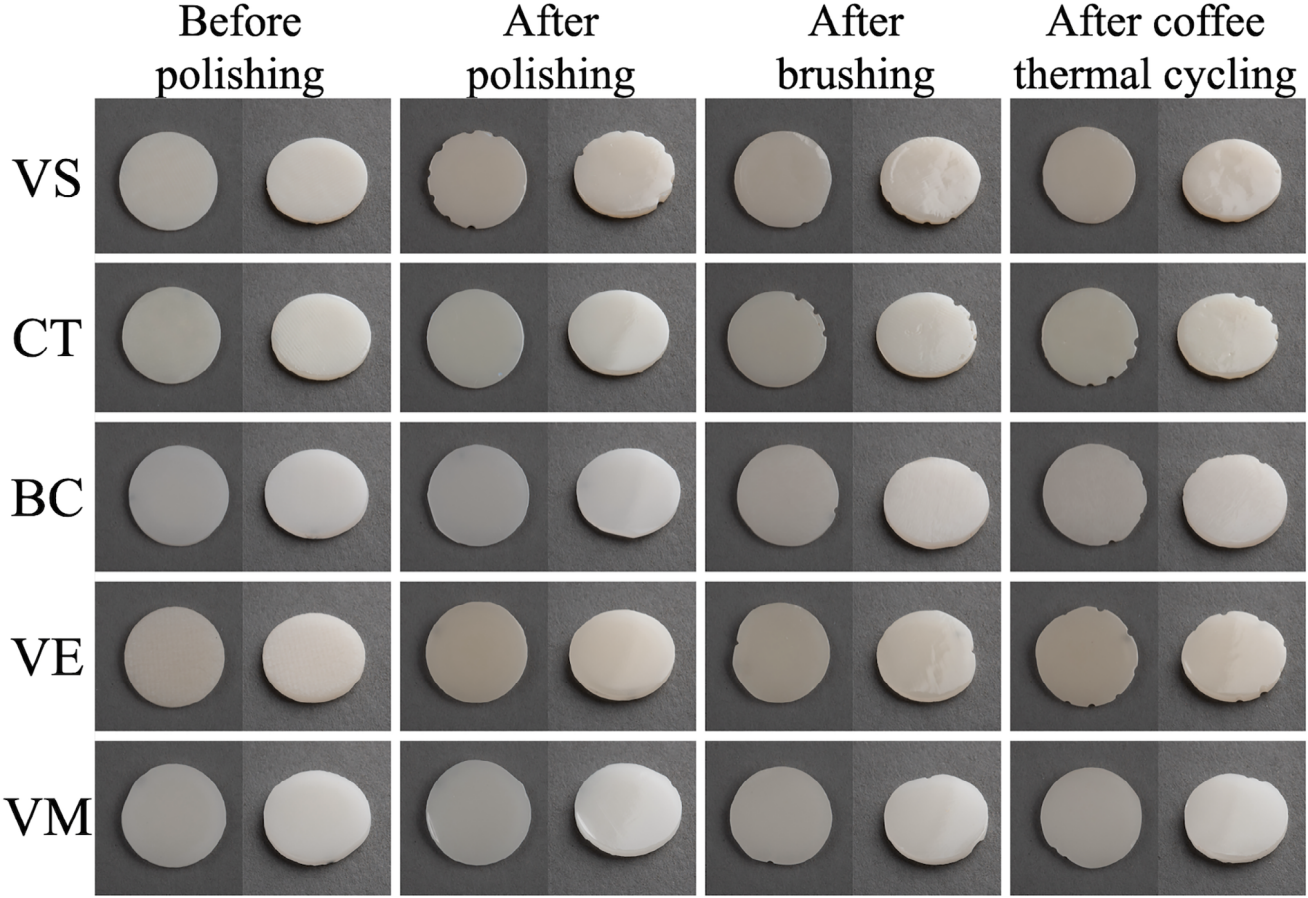
Representative image of one specimen from each material after each time interval. BC, BRILLIANT Crios; CT, CROWNTEC; VE, enamic; VM, Mark II, VS, VarseoSmile Crown Plus.

The specimens were then subjected to 10000 thermal cycles (SD Mechatronik Thermocycler; SD Mechatronik GmbH) at 5°C‐55°C in a coffee solution with a dwell time of 30 s and a transfer time of 10 s.^26^ A tablespoon of coffee (Intenso Roasted and Grounded, Kaffeehof GmbH, Bremen, Germany) was dissolved in 177 mL of water to prepare the filtered coffee solution, which was freshly made every 12 h.^1^, ^26^, ^34^ After coffee thermal cycling, to clean the coffee extracts, the specimens were brushed 10 times with toothpaste (Nevadent Complex 3; DENTAL‐Kosmetik GmbH) under running water and ultrasonically cleaned in distilled water for 10 min and dried (Figure 2).

Surface roughness, color coordinate, and microhardness measurements were repeated after brushing and after coffee thermal cycling. CIEDE2000 color difference formula with parametric factors (KL, KC, and KH) set to 1^1^, ^26^, ^34^ was used to calculate color difference (ΔE_00_) values with the coordinates measured on a gray background. The intrinsic error of the spectrophotometer (the highest ΔE_00_ value of 4 specimens within each material) ranged between 0.12 units (VM) and 0.23 units (VS). The coordinates measured on white and black backgrounds were used to calculate the translucency (RTP) of each specimen.

Shapiro‐Wilk tests revealed a non‐normal distribution for all data except for the data for microhardness. Therefore, parametric tests were used for the comparisons of microhardness values. Robust analysis of variance test was used to evaluate surface roughness, ΔE_00_, and RTP data, while a generalized linear model was used to evaluate microhardness data. Multiple comparisons were further evaluated by Bonferroni corrected post‐hoc tests. All analyses included material type and time intervals as main factors along with the interaction between main factors, and were performed with software (Jamovi v2.3.2; The Jamovi Project) at a significance level of α = 0.05. ΔE_00_ values and changes in RTP values (ΔRTP) were further evaluated for perceptibility and acceptability based on previously reported thresholds (ΔE_00_ perceptibility: 0.8 units, acceptability: 1.8 units;^40^ ΔRTP perceptibility: 0.62 units, acceptability: 2.62 units).^41^

## RESULTS

Material type affected all parameters (*p* < 0.001) other than surface roughness (*p* = 0.051), while time interval affected surface roughness and microhardness values (*p* < 0.001). However, the time interval's effect on ΔE_00_ (*p* = 0.051) and RTP (*p* = 0.270) values was nonsignificant. In addition, the interaction between the main factors affected all tested parameters (*p* ≤ 0.002). For VS, VE, and VM before polishing roughness values were the highest (*p* ≤ 0.026), and the differences among remaining time intervals were nonsignificant (*p* ≥ 0.651). For CT, the differences among remaining time intervals were nonsignificant (*p* ≥ 0.113). For BC, after brushing and after coffee thermal cycling roughness values were similar (*p* = 0.822) and higher than those of after polishing (*p* ≤ 0.012). In addition, after coffee thermal cycling roughness values were higher than those of before polishing (*p* = 0.012). Before polishing, VS had higher roughness than all materials (*p* ≤ 0.001), other than CT (*p* = 0.822). In addition, VE had higher values than those of BC (*p* = 0.001). After polishing, the differences among materials were nonsignificant (*p* ≥ 0.166). After brushing, CT and BC had similar values (*p* = 0.822) that were higher than those of VE and VM (*p* ≤ 0.021). After coffee thermal cycling, BC had higher roughness than all materials (*p* ≤ 0.010) other than VS (*p* = 0.115) (Table 3).

**TABLE 3:** Descriptive statistics of surface roughness (μm) values of each material‐time interval pair.

	Before polishing		After polishing		After brushing		After coffee thermal cycling	
Materials	Mean ± standard deviation	Median	Mean ± standard deviation	Median (Min‐Max)	Mean ± standard deviation	Median (Min‐Max)	Mean ± standard deviation	Median (Min‐Max)
VS	3.58 ± 1.01	3.59^bC^(2.03–5.52)	0.36 ± 0.12	0.33^aA^(0.22–0.55)	0.85 ± 0.47	0.85^aAB^(0.33–1.97)	0.43 ± 0.16	0.43^aAB^(0.24–0.76)
CT	2.79 ± 1.10	2.85^aABC^(1–4.51)	0.71 ± 0.62	0.51^aA^(0.34–2.43)	0.61 ± 0.14	0.62^aB^(0.42–0.84)	0.40 ± 0.11	0.38^aA^(0.25–0.57)
BC	0.27 ± 0.03	0.27^abA^(0.23–0.32)	0.15 ± 0.05	0.15^aA^(0.09–0.21)	0.81 ± 0.14	0.83^bcB^(0.64–1)	0.74 ± 0.14	0.75^cB^(0.44–0.94)
VE	0.64 ± 0.32	0.58^bB^(0.35–1.51)	0.25 ± 0.04	0.24^aA^(0.2–0.34)	0.27 ± 0.06	0.28^aA^(0.17–0.34)	0.20 ± 0.03	0.21^aA^(0.14–0.23)
VM	0.70 ± 0.11	0.64^bAB^(0.59–0.86)	0.17 ± 0.05	0.16^aA^(0.13–0.27)	0.14 ± 0.03	0.13^aA^(0.1–0.22)	0.17 ± 0.03	0.18^aA^(0.14–0.24)

Abbreviations: BC, BRILLIANT Crios; CT, CROWNTEC; VE, enamic; VM, Mark II; VS, VarseoSmile Crown Plus.

^*^Different superscript letters indicate significant differences (Uppercase letters for columns and lowercase letters for rows) (*p*<0.05).

**FIGURE 3:**
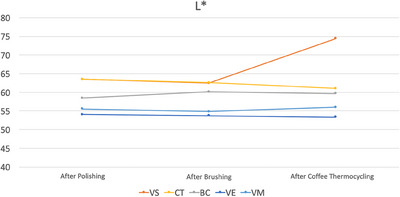
L* values of each material after each procedure. BC, BRILLIANT Crios; CT, CROWNTEC; VE, enamic; VM, Mark II, VS, VarseoSmile Crown Plus.

**FIGURE 4:**
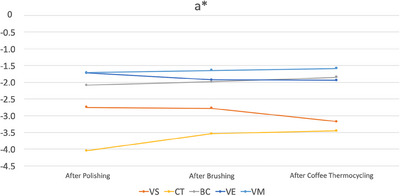
a* values of each material after each procedure. BC, BRILLIANT Crios; CT, CROWNTEC; VE: enamic; VM, Mark II, VS, VarseoSmile Crown Plus.

**FIGURE 5:**
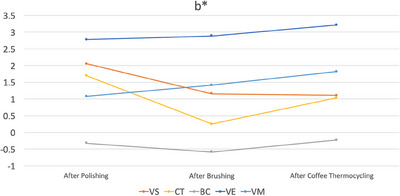
b* values of each material after each procedure. BC, BRILLIANT Crios; CT, CROWNTEC; VE, enamic; VM, Mark II, VS, VarseoSmile Crown Plus.

Among the materials tested, only VS had significantly different ΔE_00_ values among different time intervals as it had the lowest ΔE_00_ values after brushing (*p* < 0.001). After brushing, CT had higher ΔE_00_ values than VE (*p* = 0.021). After coffee thermal cycling, VS had the highest ΔE_00_ values (*p* < 0.001), while CT and VM had higher values than those of BC and VE (*p* ≤ 0.004). When all procedures were completed, VS had the highest ΔE_00_ values (*p* < 0.001). In addition, CT had higher ΔE_00_ values than those of VE and VM (*p* ≤ 0.016) (Table 4). Figures 3, 4, 5 illustrate the differences in color coordinates at each time interval and changes in these coordinates between time intervals.

**TABLE 4:** Descriptive statistics of ΔE_00_ values of each material‐time interval pair.

	After polishing versus after brushing		After brushing versus after coffee thermal cycling		After polishing versus after coffee thermal cycling	
Materials	Mean ± standard deviation	Median (Min‐Max)	Mean ± standard deviation	Median (Min‐Max)	Mean ± standard deviation	Median (Min‐Max)
VS	1.19 ± 0.68	1.36^aAB^(0.19–1.95)	9.35 ± 0.54	9.40^bC^(8.28–9.99)	9.13 ± 1.41	8.82^bC^(7.29–12.49)
CT	1.74 ± 0.52	1.85^aB^(0.75–2.32)	1.47 ± 0.46	1.37^aB^(0.90–2.49)	2.44 ± 0.52	2.40^aB^(1.67–3.32)
BC	1.27 ± 0.55	1.42^aAB^(0.41–1.91)	0.47 ± 0.20	0.39^aA^(0.31–0.94)	1.14 ± 0.59	1.15^aAB^(0.31–1.97)
VE	0.60 ± 0.27	0.60^aA^(0.15–0.95)	0.51 ± 0.42	0.40^aA^(0.12–1.58)	0.89 ± 0.30	0.90^aA^(0.38–1.45)
VM	0.87 ± 0.40	0.73^aAB^(0.27–1.67)	1.25 ± 0.29	1.31^aB^(0.82–1.70)	0.92 ± 0.14	0.92^aA^(0.73–1.21)

Abbreviations: BC, BRILLIANT Crios; CT, CROWNTEC; VE: enamic, VM: Mark II, VS, VarseoSmile Crown Plus.

^*^Different superscript letters indicate significant differences (Uppercase letters for columns and lowercase letters for rows) (*p*<0.05).

RTP values of only VS differed significantly among different time intervals as it had the highest values after coffee thermal cycling (*p* ≤ 0.049). After polishing, VM had higher RTP than those of other materials (*p* ≤ 0.002), except for VE (*p* = 0.856). Also, VS had the lowest (*p* ≤ 0.006) RTP values. After brushing, VM and VE had higher RTP values than VS and BC, while VM also had higher values than CT (*p* < 0.001). In addition, CT had higher RTP values than VS (*p* < 0.001). After coffee thermal cycling, VM and CT had higher RTP than VS and BC (*p* ≤ 0.016) (Table 5).

**TABLE 5:** Descriptive statistics of RTP values of each material‐time interval pair.

	After polishing		After brushing		After coffee thermal cycling	
Materials	Mean ± standard deviation	Median (Min‐Max)	Mean ± standard deviation	Median (Min‐Max)	Mean ± standard deviation	Median (Min‐Max)
VS	20.79 ± 1.79	20.48^aA^(17.3–23.48)	21.53 ± 1.95	21.33^aA^(17.88–24.99)	24.65 ± 1.65	24.69^bA^(21.04–27.64)
CT	25.88 ± 2.26	25.79^aB^(23.18–31.43)	25.16 ± 1.18	25.39^aBC^(22.34–26.38)	26.40 ± 0.91	26.55^aB^(24.43–27.65)
BC	25.68 ± 0.41	25.65^aB^(25.13–26.48)	24.22 ± 1.12	24.20^aAB^(22.69–26.54)	25.13 ± 0.67	25.05^aA^(24.43–26.43)
VE	30.66 ± 2.91	31.13^aBC^(25.69–34.67)	31.29 ± 3.30	32.32^aCD^(23.89–34.17)	32.04 ± 2.99	33.19^aAB^(27.34–35.46)
VM	28.84 ± 1.99	29.10^aC^(24.44–31.53)	30.04 ± 1.25	30.09^aD^(28.53–32.17)	28.37 ± 0.95	28.19^aB^(26.91–30.35)

Abbreviations: BC, BRILLIANT Crios; CT, CROWNTEC; VE, enamic; VM, Mark II, VS, VarseoSmile Crown Plus.

^*^Different superscript letters indicate significant differences (Uppercase letters for columns and lowercase letters for rows) (*p*<0.05)

Regardless of the time interval, VS and CT had similar microhardness values (*p*>0.05) that were the lowest among tested materials (*p* < 0.001). The remaining materials’ microhardness values were listed as VM, VE, and BC in decreasing order, regardless of the time interval (*p* < 0.001). Time intervals only affected the microhardness of VM, as it had the lowest microhardness after coffee thermal cycling (*p* < 0.001) (Table 6).

**TABLE 6:** Mean ±standard deviation microhardness (weight/area of indentation) values of each material‐time interval pair.

Materials	After polishing	After brushing	After coffee thermal cycling
VS	34.57 ± 1.23^Aa^	33.26 ± 1.62^Aa^	32.47 ± 1.78^Aa^
CT	30.59 ± 2.95^Aa^	29.74 ± 2.66^Aa^	30.49 ± 3.91^Aa^
BC	82.2 ± 7.08^Ba^	80.26 ± 6.81^Ba^	73.76 ± 4.69^Ba^
VE	286.3 ± 22.87^Ca^	282 ± 13.14^Ca^	266.47 ± 19.72^Ca^
VM	680.55 ± 37.73^Db^	679.93 ± 28.32^Db^	558.66 ± 39.82^Da^

Abbreviations: BC, BRILLIANT Crios; CT, CROWNTEC; VE, enamic; VM, Mark II, VS, Varseo Smile Crown Plus.

^*^Different superscript uppercase letters indicate significant differences in columns, while different superscript lowercase letters indicate significant differences in rows (*p*<0.05).

## DISCUSSION

Even though material type did not affect the surface roughness values, time interval affected measured roughness values, which led to the rejection of the first null hypothesis. In addition, the effect of material type was marginally insignificant (*p* = 0.051); thus, its effect may also be considered significant. Even though none of the tested materials had mean surface roughness values below the clinical threshold value of 0.2 μm^30^ before polishing, polishing reduced the surface roughness of all materials with some being statistically significant (VS, VE, and VM) and some being below 0.2 μm (BC and VM). In addition, the mean surface roughness value of VE after polishing (0.25 μm) can also be considered acceptable as a difference of 0.05 μm may be clinically imperceptible. For surface roughness values of materials after brushing and after coffee thermal cycling, VM constantly had mean lower values than 0.2 μm, while VE had a maximum mean surface roughness value of 0.27 μm. Therefore, it can be hypothesized that VM and VE were more resistant to consecutive brushing and coffee thermal cycling compared with other materials. A possible explanation may be the fact that VM was the only material that did not have a resin matrix, and VE had the highest ceramic filler content among the other materials. VS and CT had mean surface roughness values above the clinically acceptable threshold after polishing, which may indicate their low polishability compared with other materials. In addition, these materials, along with BC, had surface roughness values that were either similar to or higher than those of other materials and the clinically acceptable threshold after brushing and after coffee thermal cycling. These results may be interpreted as VS, CT, and BC are more prone to plaque accumulation and possibly increased antagonist wear in the long‐term given their coarser surface.

The second and the third null hypotheses of the present study were also rejected as stainability and translucency of materials were affected by material type. In addition, the effect of time interval on stainability was marginally insignificant (*p* = 0.051). When ΔE_00_ values of each material‐time interval pair were further evaluated according to previously reported threshold values,^40^ it was observed that only additively manufactured specimens had unacceptable color changes. VS had perceptible color change after brushing (ΔE_00_ = 1.19 units), while its color change was above the acceptability threshold after coffee thermal cycling (ΔE_00_ = 9.35 units) and after all procedures were completed (ΔE_00_ = 9.13 units). CT had perceptible color change after brushing (ΔE_00_ = 1.73 units) and after coffee thermal cycling, while it had unacceptable color change after all procedures were completed (ΔE_00_ = 2.44 units). Even though both materials had similar chemical compositions, slight differences between them may have led to higher susceptibility of VS to coffee thermal cycling and CT to brushing. As for the subtractively manufactured specimens, perceptible color changes were observed after brushing for BC and VM (ΔE_00_≤0.87 units), after coffee thermal cycling for VM (ΔE_00_ = 1.25 units), and after all procedures completed for all materials (ΔE_00_≤1.14 units). However, none of the subtractively manufactured materials had an unacceptable color change. Changes in color coordinates may also be associated with clinically unacceptable color changes of additively manufactured specimens as VS had increased lightness (L* values) after coffee thermal cycling (Figure 3) and CT had increased redness (a* values) after brushing (Figure 4), which were relatively consistent within other groups. Yellowness (b* values) of materials was the most affected by testing procedures (Figure 5). Brushing reduced the yellowness of additively manufactured and BC specimens, while coffee thermal cycling increased the yellowness of CT and BC, and decreased that of VS. Consecutive brushing and coffee thermal cycling increased the yellowness of VE and VM constantly.

Among the materials tested, VS had increased RTP values after coffee thermal cycling, which was unacceptable (mean ΔRTP = 3.12). In addition, consecutive brushing led to a perceptible increase in RTP for VS (mean ΔRTP = 0.74 units) and the increase after all procedures were completed was also unacceptable (mean ΔRTP = 3.86 units). As for the other materials, the highest mean ΔRTP value was 2.06 units (VE), which was only perceptible. Based on these results, it can be stated that tested subtractively manufactured materials were more resistant to discoloration and translucency change caused by combined brushing and coffee thermal cycling than additively manufactured composite resins, while VS was more prone to optical changes than CT. Nevertheless, it should also be emphasized that there was no clear trend regarding the ΔE_00_ and RTP values within materials or time intervals. This may be associated with the different chemical compositions and manufacturing methods of tested materials. However, given that the present study was the first on the combined effect of simulated brushing and coffee thermal cycling on tested materials, this hypothesis needs to be supported by future studies that investigate a broader range of materials with similar chemical compositions and manufacturing methods to the materials tested in the present study.

The fourth null hypothesis was rejected as microhardness values were affected by tested material types and time intervals. Subtractively manufactured specimens had higher microhardness values than those of additively manufactured specimens regardless of the time interval. This favorable result of subtractively manufactured specimens may be related to the fact that they were prepared by using blocks that were fabricated under controlled and standardized conditions.^11^, ^12^ Chemical compositions may also be related to the differences among tested materials and subtractively manufactured materials’ resistance to brushing. VM only had ceramic fillers and had the highest microhardness values, regardless of the time interval. Other than VM, all materials had polymeric structures as inorganic fillers, and increased ceramic filler content led to higher microhardness values. These findings are in line with previous studies.^3^, ^4^, ^9^, ^13^, ^14^ Additively manufactured specimens had the lowest microhardness values, regardless of the time interval. These results may be interpreted as the susceptibility of tested additively manufactured materials to surface degradation after physical or thermal stresses and these materials might be more prone to complications in the long term, which substantiate the results of the surface roughness tests. Nevertheless, given that higher microhardness values might result in higher antagonist wear,^4^ future studies should investigate the 2‐body wear of tested materials to elaborate these findings. Even though coffee thermal cycling only reduced the microhardness of VM, the authors think that this effect could be clinically negligible given the clinically nonsignificant mean roughness values after coffee thermal cycling.

The number of specimens in the present study was similar to those in previous studies with resembling methodology,^6^, ^9^, ^30^, ^31^, ^32^ and significant differences were found among tested materials and time intervals for each parameter investigated. In addition, post hoc power analyses were performed for each parameter investigated and the sample size was deemed adequate for a minimum of 78% power with a minimum effect size of 0.48 and α = 0.05 for those groups with statistically significant differences within the investigated parameters. Nevertheless, the absence of a priori power analysis is a limitation of the present study. Another limitation of the present study was that all materials were prepared at a certain thickness in a single shade; both parameters may affect color and translucency.^22^ Simulated brushing test had standardized parameters to reflect nearly 7 years of brushing; however, differences in the load applied, frequency of brushing, the abrasiveness of the toothpaste, microhardness of the bristles of the toothbrush, and the dilution of the toothpaste may affect these results. The coffee thermal cycling may have led to discoloration of both surfaces of the specimens and amplified color change, as clinically, only polished or glazed surfaces are exposed to beverages.^34^ The aging duration can be considered excessive as 10000 cycles attempt to simulate coffee consumption over many years, and the test set‐up potentially represents a worst‐case scenario. The clinical service of restorations made of tested materials may resist longer durations of staining for individuals who don't consume discoloring fluids such as coffee. In addition, even though coffee was reported to accelerate discoloration due to its acidic components,^8^ different staining solutions may alter the results.^16^ The profilometer and spectrophotometer used in the present study have been used in previous studies.^30^, ^34^ However, given the variety of devices that can be used to measure surface roughness and color coordinates, it should be noted that different instruments may change the results. In addition, color threshold values used in the present study vary across studies and different interpretations can be made if other published threshold values are used. Finally, because the present study was the first on the combined effect of long‐term brushing and coffee thermal cycling on the properties of additively manufactured composite resins indicated for definitive prostheses, comparisons with previous studies were not possible. Therefore, the results of the present study should be corroborated with future in vitro studies on how these processes affect different mechanical properties and with in vivo studies that focus on how these processes affect the clinical stability and longevity of tested additively manufactured composite resins.

## CONCLUSIONS

Polymer‐infiltrated ceramic network and feldspathic ceramic surfaces were more resistant to roughening with long‐term brushing and coffee thermal cycling. Additively manufactured composite resins had higher unacceptable surface roughness and were more prone to discoloration and translucency change after all procedures were completed. Tested additively manufactured composite resins had the lowest microhardness and feldspathic ceramic had the highest microhardness. Brushing did not affect the microhardness of tested materials and coffee thermal cycling only reduced the microhardness of tested subtractively manufactured feldspathic ceramic. Based on the parameters tested, restorations fabricated by using tested feldspathic ceramic may have a higher clinical stability and lesser probability of surface‐related and esthetic complications.

## CONFLICT OF INTEREST STATEMENT

The authors have no conflict of interest.
